# What is required to facilitate implementation of Swedish physical activity on prescription? – interview study with primary healthcare staff and management

**DOI:** 10.1186/s12913-018-3021-1

**Published:** 2018-03-21

**Authors:** Catharina Gustavsson, Maria Nordqvist, Kristina Bröms, Lars Jerdén, Lena V. Kallings, Lars Wallin

**Affiliations:** 1 Center for Clinical Research Dalarna, Department of Public Health and Caring Sciences, Uppsala University, Nissers vag 3, SE-79182 Falun, Uppsala Sweden; 2 School of Education, Health and Social Studies, Dalarna University, SE-79188 Falun, Sweden; 3 School of Health, Care and Social Welfare, Mälardalen University, Box 883, SE- 721 23 Västerås, Sweden; 4 Department of Public Health and Caring Sciences, Family Medicine and Preventive Medicine, Uppsala University, BMC, Box 564, SE-751 22 Uppsala, Sweden; 5 Centre for Research & Development, Uppsala University, Region Gävleborg, CFUG, SE-801 88 Gävle, Sweden; 6 Department of Sport and Health Sciences, Swedish School of Sport and Health Sciences (GIH), Box 5626, SE-114 86 Stockholm, Sweden; 7 Department of Neurobiology, Care Sciences and Society, Division of Nursing, Karolinska Institutet, SE-177 70 Stockholm, Sweden; 8 Institute of Health Care Sciences, Sahlgrenska Academy, University of Gothenburg, Box 457, 40530 Gothenburg, Sweden

**Keywords:** Disease prevention, Health professionals, Health promotion, Implementation, Non-communicable disease prevention, Physical activity, Primary healthcare, Qualitative research method, Sweden

## Abstract

**Background:**

The method, Swedish Physical Activity on Prescription (SPAP), has been launched in Swedish healthcare to promote physical activity for prevention and treatment of lifestyle related health disorders. Despite scientific support for the method, and education campaigns, it is used to a limited extent by health professionals. The aim of the study was to describe the views of health professionals on perceived facilitators, barriers and requirements for successful implementation of SPAP in primary healthcare.

**Methods:**

Eighteen semi-structured interviews with stakeholders in SPAP, i.e. ten people working in local or central management and eight primary healthcare professionals in two regional healthcare organisations, were analysed using qualitative content analysis.

**Results:**

We identified an overarching theme regarding requirements for successful implementation of SPAP: *Need for knowledge and organisational support*, comprising four main categories: Need for increased knowledge and affirmative attitude among health professionals; Need for clear and supportive management; Need for central supporting structures; Need for local supporting structures. Knowledge of the SPAP method content and core components was limited. Confidence in the method varied among health professionals. There was a discrepancy between the central organisation policy documents declaring that disease preventive methods were prioritised and a mandatory assignment, while the health professionals asked for increased interest, support and resources from management, primarily time and supporting structures. There were somewhat conflicting views between primary healthcare professionals and managers concerning perceived barriers and requirements. In contrast to some of the management’s beliefs, all primary healthcare professionals undisputedly acknowledged the importance of promoting physical activity, but they lacked time, written routines and in some cases competence for SPAP counselling.

**Conclusion:**

The study provides knowledge regarding requirements to facilitate the implementation of SPAP in healthcare. There was limited knowledge among health professionals regarding core components of SPAP and how to practise the method, which speaks for in-depth training in the SPAP method. The findings highlight the importance of forming policies and guidelines and establishing organisational supporting structures, and ensuring that these are well known and approved in all parts of the healthcare organisation.

**Electronic supplementary material:**

The online version of this article (10.1186/s12913-018-3021-1) contains supplementary material, which is available to authorized users.

## Background

Regular physical activity has a positive impact on both mental and physical health. Yet, almost half of adults in high income countries are insufficiently physically active [1]. Physical inactivity is a public health threat and one of the ten most significant risks factors for loss of disability adjusted life years worldwide [2].

Healthcare services have a unique role in promoting health, as it reaches a large part of the population, including vulnerable groups which may otherwise be difficult to reach, such as the elderly, socio-economically weak and people on sick leave [3]. Guidelines in Swedish healthcare regarding methods for preventing diseases caused by unhealthy lifestyle behaviours, such as tobacco use, harmful use of alcohol, physical inactivity and unhealthy diet, were launched by the Swedish National Board of Health and Welfare in 2011 [4]. These guidelines [4] recommend that in regard to insufficient physical activity, healthcare should offer person-centred health promotion counselling, combined with written prescription of physical activity or monitoring of physical activity by pedometers, and also a follow-up of the prescribed physical activity.

The Swedish Physical Activity on Prescription (SPAP) was introduced in 2001, as a method for Swedish healthcare to promote physical activity both for the prevention and treatment of lifestyle related health disorders [5]. The SPAP method consists of five core components: 1) person-centred health promotion consultation, 2) written prescription of physical activity summing an agreement between the patient and the health professional based on the expressed intentions and goals of the patient, and the knowledge and competence of the health professional, 3) a prescription guided by evidence-based knowledge on physical activity in the prevention and treatment of health conditions; i.e. in accordance with the recommendations in the handbook “Physical Activity in the Prevention and Treatment of Disease FYSS” [6], 4) follow-up of the written prescription, and 5) collaboration between the healthcare service and physical activity organisers outside healthcare (e.g. sports clubs, fitness centres). It is also emphasised that the method should be tailored to local conditions in the healthcare organisation [7, 8].

Studies have reported that prescriptions in line with SPAP increase physical activity level in patients in primary healthcare [9, 10]. SPAP has also been shown to reduce sedentary time [11], have a positive effect on health-related quality of life [9, 12] and on risk factors for metabolic syndrome and cardiovascular diseases [11, 13].

Despite scientific support for SPAP and its components, the use in healthcare services has been low [4, 14]. Since the introduction, attempts have been made to facilitate implementation of SPAP in primary healthcare practice, mainly through information and the education of healthcare professionals. However, these attempts have not yielded a lasting effect on the usage of the method [8]. There are few studies on how the components of SPAP are applied clinically, or on how to support implementation of SPAP in healthcare services [15–17].

Implementation of new healthcare practices can be seen as a process of behavioural change that is affected by many actors and factors on different levels [18]. Health promotion practices often take a longer time to implement in clinical practice as compared to, e.g. new technologies [19, 20]. According to The Promoting Action on Research Implementation in Health Services framework (PARIHS), successful implementation is a function of the interaction of: *evidence*, i.e. the degree of scientific support for the proposed change, *context*, i.e. the environment in which the proposed change is to be implemented, and *facilitation*, i.e. the manner in which the change is supported [18, 21]. Factors of importance for successful implementation of a new method include the individual healthcare provider’s attitude to the method, and that it targets a perceived need by the users as well as general features, such as the organisational culture. The implementation strategy should involve a clearly structured method for the clinical work, and the application of the method should fit as part of regular procedures. If relevant and evidence-based methods are not applied in healthcare, it is important to identify the underlying mechanisms for non-application, in that appropriate strategies to support implementation can be developed [20]. It would also be important to identify the personal and contextual barriers and facilitators for implementation of the SPAP method in healthcare, in order to gain better understanding of factors affecting the process. Thus, the aim of the study was to describe the views of health professionals on perceived facilitators, barriers and requirements for successful implementation of SPAP in primary healthcare.

## Methods

### Study design

This is a descriptive study using qualitative content analysis [22] of interview data with both a deductive and inductive approach [23].

### Participants

We used a purposeful sampling approach to select stakeholders in SPAP in two regional healthcare organisations in Sweden. The participants consisted of ten people working in primary healthcare management (three managers of healthcare centres; two local SPAP coordinators; and two managers and three health promotion coordinators in the central administration of the healthcare organisations), and eight health professionals working in primary healthcare centres (three physicians, two registered nurses and three physiotherapists). In all, eighteen stakeholders were interviewed: five stakeholders at management level from each region plus five and three health professionals from each region respectively. The sampling was done to represent maximal variation in type of profession, management level, number of SPAP prescriptions at the primary healthcare centres and the size of the primary healthcare centres. By including the two regional healthcare organisations it was possible to illuminate differences with regard to the organisation of health promotion services, in that one organisation provided central guidelines, other materials and advisory support on SPAP to the healthcare staff to a much greater extent than the other organisation.

### Ethical considerations

The study was approved by The Ethics Review Board at Uppsala University (EPN Uppsala No. 2014–198). Verbal and written information about the study were provided to the participants. Written consent for participation were obtained from the participants prior to the interviews.

### Procedure

Individual interviews were undertaken during the autumn of 2014 according to a semi-structured interview protocol [24]. The protocol contained open ended questions designed to respond to the research questions of the study: What factors in the organisational context facilitate or impede prescription of SPAP in patient consultations? What requirements are needed to facilitate increased prescription of SPAP? During the interview, the interviewer posed follow-up questions, restated and summarised information, and asked the participant to confirm the accuracy of the spoken data [25]. English language copies of the two Interview Guides used to direct discussions are attached as a supplementary file (Additional file 1). The interviews lasted for 15–45 min and were carried out in a room at the participant’s place of work where only the participant and the interviewer were present and undisturbed by others. The second author conducted all of the interviews of participants in management positions, and a research assistant (a physiotherapist working in primary healthcare and well acquainted with the SPAP method), conducted all of the interviews with the health professionals. The interviews were audio-recorded and transcribed verbatim directly after each interview by a research assistant.

### Data analysis

The interview data was analysed using qualitative content analysis [22], and undertaken in two steps [23]. First, the interviews with the participants working in healthcare management were analysed by an inductive approach. Second, the interviews with health professionals were analysed by a deductive approach based on the categorisation matrix created from the healthcare management interviews.

The first and the second author read the text files of the interviews as soon as they had been transcribed in order to gain an overview of the material, along with a sense of when the material was sufficiently saturated, i.e. when similar descriptions of attitudes and perceptions of facilitators and barriers to the use of SPAP recurred in the interviews.

The first and the second author also performed the initial data analysis by reading the text and identifying meaning units, i.e. specific units of text consisting of a single word, a few words, or a few sentences relating to the research questions. Meaning units were condensed and coded. Codes were then discussed and grouped together into higher order subcategories and categories. In order to minimise bias, a dialogue was ongoing between the two authors, which facilitated openness to the text with its meaning units, codes, subcategories and categories. In order to validate the interpretation, all authors read the text files, discussed the coding and reviewed the preliminary interpretation. All authors participated in the analysis by discussing and revising the interpretation until consensus was obtained. Table 1 provides examples of the analytic coding process by which meaning units, codes, subcategories and categories were formed.

**Table 1:** Subcategories and examples of codes and meaning units in the category “Need for central supporting structures”

Category	Subcategories	Examples of codes	Examples of meaning units
Need for central supporting structures	Establish a centralised function within the healthcare organisation for coordination and development of SPAP	Support (by education, written material) from central health promotion unit	*...someone who lectured and talked about the method at the healthcare centres, if there were experts who could come out to us.*
			*...you need to get more information out, to us here at the primary healthcare centre, on how to use this method…*
		Support (by inspiration, coaching and regular updates) from central health promotion unit	*...some sort of events or something that happens all the time, so that you could get inspiration...*
			*...we used to get to meet the central health promotion coordinator once or twice a year for updates on our prescriptions and for some inspiration*
	Expand cooperation with external physical activity organisers	Central support for organised cooperation outside healthcare	*...someone who finds clever ways when working with other organisations* [i.e. outside healthcare] *and referring patients.*
			*...someone, like a central coordinator or something, so that updating* [of contact list to activity organisers] *is done all the time*

*SPAP* Swedish Physical Activity on Prescription

Open coding using an inductive approach was used for the data generated by the interviews with those working in healthcare management positions. After coding, the data from the interviews with health professionals was grouped into the pre-defined subcategories and categories in the structured categorisation matrix created from the inductive analysis. When using this matrix we selected the aspects (meaning units) that fitted the categorisation frame, but were still open to that aspects which did not fit the categorisation could be used to create new categories to be added to the matrix.

## Results

The content analysis identified several requirements for successful implementation of SPAP and generated the overarching theme: *Need for knowledge and organisational support* comprising four main categories: Need for increased knowledge and affirmative attitude among health professionals; Need for clear and supportive management; Need for central supporting structures; Need for local supporting structures. The analysis of interviews with health professionals did not reveal any new categories as compared to the categorisation matrix created from the healthcare management interviews. The categories and subcategories are described in Table 2 and in further detail below, with quotations in italics.

**Table 2:** Requirements for successful implementation of SPAP. Qualitative content analysis of interviews with healthcare stakeholders (*n* = 18)

Overall theme: Need for knowledge and organisational support	
Categories	Subcategories
Need for increased knowledge and affirmative attitude among the healthcare professionals	Increase knowledge on how to talk about health behaviours in patient consultations
	Increase knowledge and belief in the SPAP method
	Increase understanding of the importance of targeting and supporting physical activity in healthcare
Need for clear and supportive management	Establish policies and clinical guidelines, and make them well known and approved by all parts of the organisation
	Increase interest and support from the management
	More resources, primarily time, earmarked for conducting SPAP in patient consultations in daily clinical practice
Need for central supporting structures	Establish a centralised function within the healthcare organisation for coordination and development of SPAP
	Expand cooperation with external physical activity organisers
Need for local supporting structures	Develop locally tailored routines for how to work with SPAP at each healthcare centre
	Appoint a specialised function: “local SPAP coordinator”, at each healthcare centre

*SPAP* Swedish Physical Activity on Prescription

### Need for increased knowledge and affirmative attitude among health professionals

#### Increase knowledge on how to talk about health behaviours in patient consultations

All participants perceived that it was important to address health behaviours in patient consultations/encounters, but were not sure that their colleagues felt it was important. Specifically, some of the participants in central management positions perceived that the health professionals felt that it was not their duty to undertake health promotion.

*“...even now, it is still not natural for most of the Western world healthcare to work that way. I mean, to allow patients to take more responsibility.”*

However, several of the health professionals stated that, although they wanted to, they did not have enough competence in undertaking health promotion consultations, and/or that they felt unaccustomed to communicate health behaviours. Some of the health professionals had attended courses in motivational interviewing and “quit-smoking” consultations, but they had seldom applied the knowledge to their current practice.

*“…and to address healthy lifestyle requires that one feels confident in that type of questions and knows what to do with the answers and so on.”*

#### Increase knowledge and belief in the SPAP method

There was limited knowledge among the participants regarding the content of the SPAP method and how to use the components of SPAP in clinical practice. Very few of the participants had in-depth knowledge on the SPAP method, i.e. neither the rational and theoretical underpinnings for the method, nor the content and mode of use of all the core components. For most of the participants, the written prescription, i.e. component 2, was equivalent to the SPAP method. Only a few of the participants stressed the importance of the person-centred health promotion consultation, i.e. component 1, prior to prescription. Some of the participants knew that undertaking follow-ups of the prescription, i.e. component 4, was supposed to be part of the SPAP method. A majority knew about the Physical Activity handbook FYSS [6], i.e. component 3, but only one stated using it for guidance in prescribing SPAP. The majority of the informants did not know whether they had the possibility to collaborate with physical activity organisers outside their healthcare organisation, i.e. component 5. Likewise, most participants had vague knowledge of the scientific support for the SPAP method.

One participant underlined that SPAP is a complex method requiring extensive knowledge on principles for behavioural medicine, e.g. communication for increasing motivation for behaviour change. Moreover, the participants stated that such knowledge is largely lacking among healthcare professionals.

*“It’s a problem that most people in healthcare have no knowledge on methods for behaviour change. You need to know that, to be able to understand why SPAP is good. If health professionals had better knowledge it would be much easier, that would make a difference.”*

All participants in management positions stated high confidence in SPAP. Among the interviewed health professionals, the belief in SPAP varied: some had a very positive attitude towards the method, some were hesitant and some had a negative attitude. Several participants mentioned that lack of confidence in the effectiveness of the SPAP method was a common reason for its low use among health professionals. The participants reported that overall, the belief in the importance and value of SPAP varied among employees in the healthcare organisation. The lack of confidence in the SPAP method was highlighted as an important barrier to implementation.

*“SPAP, ...a great tool to use in order to make a behaviour change.”*

*“... it's being questioned more and more. Well, I know that SFAM, the Swedish College of General Practice, has had some sort of group who has said that we should not use SPAP. I still think SPAP is very good. But of course, one has to do what is scientifically proven and I am not updated enough on this. Nevertheless, those are important people in SFAM who state: -We should not be doing this, SPAP is not good enough.”*

#### Increase understanding of the importance of targeting and supporting physical activity in healthcare

There was a total concordance in opinions on the health benefits of increased physical activity and that healthcare had a role in the promotion of physical activity. In contrast to several of the management’s beliefs, the health professionals stated that they had understanding of the importance of promoting physical activity in patient consultations. The health professionals perceived that physical activity was a health behaviour that was easy to address, as opposed to addressing food/nutrition or alcohol/drugs. Probably owing to the general opinion in society brought about by mass media on the health benefits of physical activity, many patients welcomed discussing physical activity in relation to their health.

*“…instead of pills…supporting people to take responsibility and start to move, as everybody knows, that it is much more beneficial.”*

### Need for clear and supportive management

#### Establish policies and clinical guidelines, and make them well known and approved by all parts of the organisation

The participants in central management positions in both healthcare organisations declared that they had central policy documents for applying disease preventive methods in the healthcare services. There was a discrepancy between the management stating that there were policy documents available on disease preventive methods, and most of the interviewed primary healthcare professionals being unaware of the content in those documents, and in some cases of their very existence. Many of the health professionals perceived that they did not have access to central policy documents that provided enough guidance and direction for undertaking SPAP.

*“…you don’t have an explicit instruction that you should be doing this, so it becomes a bit unclear. It’s like there is no real job description.”*

One of the healthcare organisations had previously provided reimbursement based on the number of written SPAP prescriptions. This was perceived by some participants as beneficial in increasing focus on SPAP, but perceived by others as limiting the SPAP method to the written prescription only.

*“I’m more afraid that it will be more about quantity, a lot of prescriptions are written without having had communication with the patient.”*

#### Increase interest and support from the management

The participants in central management positions in both healthcare organisations affirmed that applying disease preventive methods was a prioritised and mandatory assignment in the healthcare services, whereas many of the primary healthcare professionals called for pronounced interest and support from the management. None of the interviewed primary healthcare professionals perceived that management expressed the importance of providing health promotion counselling, nor did central management explicitly show that they prioritised health promotion. Working according to SPAP was based on personal commitment.

*“It is not central management or a boss, who says, now we're going to do this, it is dependent on the commitment from those who work, I think.”*

Many of the informants emphasised the importance of the management’s endorsement and attitude for the implementation of SPAP.

*“...when it really comes from above, saying ‘now this is the priority’, then there will be power in it.”*

#### More resources, primarily time, earmarked for conducting SPAP in patient consultations in daily clinical practice

Most participants, both at management and health professional level, described the SPAP method as time-consuming and that they often felt they did not have sufficient time to work according to the method during normal consultations. One participant at management level pointed out that, so far, cost estimates for health promotion had never been taken into account in the budget preparation in the healthcare organisation. The participants emphasised that extra money is needed to establish networks, central supporting structures and cooperation with activity organisers. Some of the participants pointed out that one way to get resources is by stopping doing other things, i.e. reallocate resources within the healthcare organisation. Giving genuine priority to SPAP would generate resources because something else would lose priority.

*“The decision has been made in our healthcare organisation that we should work with it, but they have not appointed any resources ... then of course, there’s the conflict, you should do this but you will get no more resources to do it.”*

In addition to more time, the health professionals pointed out that not having a computer and printer close by was a barrier to providing the written prescriptions.

*“We don’t have computers in the treatment rooms, so you have to stand in a corridor and produce the written prescription and talk about it with the patient.”*

Furthermore, it was difficult to find additional appointment times in the calendar for follow-ups of physical activity prescriptions.

### Need for central supporting structures

#### Establish a centralised function within the healthcare organisation for coordination and development of SPAP

To have a centralised support function, such as a central SPAP-coordinator and/or SPAP-educator, was perceived important by all participants. Both healthcare organisations had a central unit for health promotion. In one of the organisations, the unit has been well established for many years and visible to the health professionals, and in the other organisation it was not well known among the employees. Yet the participants in both organisations expressed the need for more tangible support from the central health promotion units. Having an easily available central supporting function would facilitate implementation. The participants claimed that primary healthcare management did not have the time or capability to provide such support. Providing inspiration and coaching, distributing written information and organising educational activities, plus keeping the organisation updated on changes and news, were described as appropriate tasks for such a centralised function.

*“Yes, if we could get someone who lectured and talked about the method at the healthcare centres, if there were experts who could come out to us. That way, there would be people who could help to get things started.”*

#### Expand cooperation with external physical activity organisers

Central support for organising cooperation with activity organisers outside healthcare (e.g. sports clubs, fitness centres), was expressed as important for increased physical activity, not least for transitioning the patient to physical activity organisers outside healthcare. Many of the participants underlined the limited collaboration between healthcare and physical activity organisers. Contact lists of activity organisers were available at some of the primary healthcare centres, but were not updated and therefore not helpful. Some of the participants wanted help in organising cooperation with external physical activity organisers, and suggested that updated contact lists provided by a central supporting function would be valuable.

*“…then also someone who finds clever ways of working with other organisations* [i.e. outside healthcare] *and referring patients. That has to work! And that there is someone, like a central coordinator or something, so that updating* [of contact list to activity organisers] *is done all the time.”*

### Need for local supporting structures

#### Develop locally tailored routines for how to work with SPAP at each healthcare centre

Lack of local written routines at healthcare centres was an important barrier to prescribing SPAP. Several participants stated that they had no time to find out how they were supposed to provide SPAP and therefore simply spoke to the patient about health-promoting physical activity and in some cases wrote down a few pieces of advice. The primary healthcare professionals called for written guidance on how to undertake SPAP, by written routines that could be locally tailored to conditions at each primary healthcare centre. One of the healthcare organisations had developed such documents. Several of the participants pointed out that written local routines facilitated the raising of issues of health promoting physical activity with patients and that a written local routine for SPAP was of the utmost importance in the guidance and structure of local work.

*“…I mean, clarify: how do we work with this here at our healthcare centre? How do we build our own organisation around this? Who does what? I think that is important.”*

Several participants, particularly the physicians, experienced a lack of time and expertise to undertake SPAP and expressed a need for cooperation with a physiotherapist, i.e. a health professional perceived to be an expert on physical activity for people with complex health conditions. Those primary healthcare centres that had clear written routines for cooperation and referral pathways were reported by the participants to be more successful in the implementation of SPAP.

*“One forgets that it doesn’t have to be me who prescribes, maybe I can talk with the patients about physical activity and then I send them to the physiotherapist, who is more specially trained to work with physical activity and motivation.”*

Still, the participants in management positions emphasised the importance of all health professionals sharing responsibility to advocate health promotion. Local routines for SPAP were perceived as important to clarify for all health professionals about their role and responsibilities in the health promotion process.

*“There are some parts that are everyone's responsibility…”*

*“Everyone should be able to identify these patients.”*

#### Appoint a specialised function “local SPAP coordinator”, at each healthcare centre

Many participants viewed the availability of people with expert knowledge in SPAP at the primary healthcare centre as important for implementation. Having one of the staff appointed “local SPAP-coordinator” was regarded favourable in using the SPAP method. When working well, the local coordinator was the champion for SPAP, providing reminders, advice and support to the others, thus pushing the development forward.

*“... it is necessary that there are some people who know how to do this a little bit better, I think. And who can support locally, at the primary healthcare centre. And that you really feel that support as a healthcare staff member.”*

Some of the participants reported that follow-up of physical activity prescriptions was difficult to manage. They expressed the need to have a local SPAP coordination function at each primary healthcare centre with time for follow-up activities. In addition, some of the interviewed health professionals claimed that conducting SPAP was beyond their competence, specifically where patients with health disorders including comorbidity were concerned. The physiotherapist was mentioned by several participants as particularly suitable for the role of local SPAP coordinator, as an expert on physical activity for people with complex health disorders.

## Discussion

This study disclosed key requirements for the implementation of the SPAP method in primary healthcare, in particular, the need for knowledge of the method and organisational support. An important facilitator revealed was that all health professionals undisputedly acknowledged the importance of promoting physical activity. Identified barriers for implementation were insufficient knowledge among health professionals of the core components in SPAP and lack of organisational support, including accessibility of policy documents and clinical guidelines, explicit management endorsement of SPAP and lack of resources, primarily time.

All participants had a positive attitude towards health promotion and agreed that it was important for healthcare to address health behaviours and support physical activity in patient consultations. However, only a few used the SPAP method for that purpose. This is not surprising since the basic knowledge of the method was lacking. It was an interesting finding that most participants revealed a lack of knowledge of the SPAP method: i.e. its components and theoretical underpinnings, and how to apply it. The health professionals also reported that they lacked time to use SPAP, in part referring to that the method was time consuming and due to contextual barriers, such as not having computer and printer in the same room where they met the patients. But in our interpretation this might be another way of expressing a lack of understanding of the rationale and benefits of the method and of how the core components of SPAP are intended to be applied. However, it was also clear that the health professionals felt that they did not have enough organisational support for providing SPAP, neither from policy documents and clinical guidelines, nor from management.

Our study is helpful in that only a few studies have investigated how the core components in SPAP are used clinically and how the implementation of SPAP in healthcare can be facilitated [15–17]. The findings in our study suggest that there is limited knowledge regarding the components of the SPAP method. Many of the interviewed health professionals, who are the potential users of the method, had little knowledge of the other components, apart from the written prescription. These findings are similar to the results of an interview study on patients who had been prescribed SPAP by physicians in primary healthcare [26]. Those patients perceived that they had been given very little information about SPAP, they had not been listened to and actively involved in the prescription and they were uncertain about the distinction between SPAP and physiotherapy. This corresponds well to our finding that very few of the informants stressed the importance of the person-centred health promotion consultation, i.e. component 1, prior to prescription. The widespread misconception of the rationale and components of SPAP is further shown in the criticism made by a task force in The Swedish College of General Practice, claiming that SPAP is an unnecessary and potentially harmful method that should not be used in healthcare [27]. These authors assert that “SPAP is a patriarchal method” implying that the focus is on the written prescription and that it is issued by the health professional based solely on professional competence, ignoring the patient’s involvement [27]. This criticism was based on results from other methods for physical activity promotion (i.e. exercise referral in the UK [3]), and thereby fails to recognise that the SPAP method emphasises the importance of the person-centred health promotion consultation. Nevertheless, it has had impact on practitioners as revealed in our interviews.

We suggest that understanding of the theoretical underpinnings for the components in the SPAP method is important for the motivation to apply the method. Thus, education in the SPAP method should include knowledge about behaviour learning principles, social cognitive theory [28] and communication techniques that promote behaviour change, such as Motivational Interviewing [29]. This kind of knowledge generates proficiency in addressing cognitive and behavioural risk factors for problematic health outcomes [30], and for supporting patients in goal-setting techniques and self-monitoring of health behaviours in relation to physical activity [31, 32].

Another important finding was that although central policy documents in both healthcare organisations stated that applying disease preventive methods is a prioritised and mandatory work task in healthcare services, several of the participants expressed that there were not enough local supporting structures, such as locally tailored routines and local SPAP coordinators. This was especially prominent in the healthcare organisation that had a central health promotion unit that was less known among the health professionals. More surprisingly, in the other healthcare organisation that had an active central health promotion unit, the participants still perceived that they had insufficient locally tailored routines. The expressed need for firm and substantial central support could proposedly be provided through lectures presenting content of new/updated policies and guidelines, regular alerts on updates, updating lists of physical activity organisers, and guidance in how to convert central policies and guidelines into locally tailored routines. Having elaborated central guidelines into locally tailored routines at each primary healthcare centre has been suggested as important for successful implementation [14].

Specifically, undertaking follow-up of written physical activity prescriptions was perceived difficult to manage by the health professionals due to lack of time and resources. Providing follow-up of health behaviours, such as health-promoting physical activity, has been shown to be important for adherence to the prescription and for success in health behaviour change, and thus an important component in the SPAP method [17]. Some of the informants expressed the need for a local SPAP coordination function with time to undertake follow-up of prescriptions. This corresponds in some part to the results of another Swedish study [15, 16] on general practitioners, showing that their willingness to prescribe SPAP increased if they could cooperate with a physiotherapist in the prescription process. In that study, the physicians stated that they were happy to initiate the SPAP process, but wished that the physiotherapist would complete the motivational interview and agreement with the patient regarding the type of activity, duration and intensity, and also carry out the prescription follow-up. According to Persson et al. [15, 16], the physicians felt that prescribing SPAP was neither part of their professional role, nor did they have time to prescribe and carry out follow-up of the prescription. In our study, the wish to cooperate with a physiotherapist was expressed by some participants, but not all. In contrast, some of the participants emphasised that all health professionals had a shared responsibility to advocate health promotion.

By using a qualitative method for data collection, a broad range of requirements for facilitating implementation of the SPAP method was revealed that would otherwise have been difficult to identify through a quantitative design. A purposeful sampling strategy was used in order to obtain a rich and broad material presenting maximum variation [33]. The sample was selected to include as many of the factors in participants’ characteristics and settings, which might affect variability and have relevance to the conceptualisation of the phenomenon, as possible. However, there is a risk of selection bias or an incomplete sample by such a method. In this study credibility and transferability was enhanced by using a sample of participants representing diversity of professional background, different levels in the healthcare organisations and representing two healthcare organisations with slightly different contextual conditions. Still, the small sample of participants in our study is a limitation to transferability of the result. We only interviewed two or three participants from each category of health profession. It is possible that the participants were not representative of all people with the same professional background. Some of the study findings are in correspondence with other Swedish studies, thus indicating that the result might be generalised to other parts of the Swedish primary healthcare, not solely the two healthcare organisations studied here. We only interviewed stakeholders within the healthcare organisations, and thus the perspective of the patient was not included. To reduce the risk of bias in data collection and analysis, respondent validation (member checking) was undertaken during the interviews [25]. To increase trustworthiness in the interpretation of data, all researchers read the text files of the interviews and participated in discussing the coding, categorisation and interpretation of data [24, 25].

## Conclusion

This study adds important knowledge to the use of SPAP in primary healthcare, and to the requirements that could facilitate the implementation of SPAP. There was limited knowledge among the health professionals regarding core components of the SPAP method, with the exception of the written prescription. This speaks for more in-depth education in how to practise the method, including knowledge of the rationale for SPAP and its theoretical underpinnings. The findings highlight the importance of forming policies and clinical guidelines, and establishing organisational supporting structures, as well as ensuring that these are well known and approved in all parts of the healthcare organisation. The identified key requirements for implementation will guide the development of an implementation intervention that targets these factors.

## Additional file

Additional file 1: English language copies of the two Interview Guides used to direct discussions. Interview guide 1: key stakeholders for SPAP in the healthcare organisations. Interview guide 2: prescribers of SPAP in the healthcare organisations. (DOCX 23 kb)

## Abbreviation

SPAP: The Swedish Physical Activity on Prescription
