# Supplementary material for: What is required to facilitate implementation of Swedish physical activity on prescription? – interview study with primary healthcare staff and management
Source: BMC Health Serv Res. 2018 Mar 21;18:196. doi: 10.1186/s12913-018-3021-1 (PMC5863486; doi:10.1186/s12913-018-3021-1)
Supplement: Supplementary file 1 — English language copies of the two Interview Guides used to direct discussions. Interview guide 1: key stakeholders for SPAP in the healthcare organisations. Interview guide 2: prescribers of SPAP in the healthcare organisations. (DOCX 23 kb) [file 12913_2018_3021_MOESM1_ESM.docx]

**Interview guide – key stakeholders for SPAP in the healthcare organisations**

**Introduction**: “The topic for this interview is the method Physical Activity on Prescription, which is usually shortened to SPAP. For more than 10 years, the method has been used in Swedish healthcare to increase health-promoting physical activity in the population. But the method has been used in many different ways in different county councils/healthcare organisations. I am interested in your experiences and thoughts about SPAP from an organisational perspective, ie. your thoughts on organisational factors and their significance for the use of the SPAP method in the healthcare organisation.
I will ask you some questions, the interview will be recorded, and transcribed to text after the interview. The whole interview takes about half an hour. The transcribed interviews will be coded, i.e. all information that could be linked to you will be deleted from the text. This is done to ensure that you and your answers during the interview should not be possible to recognize when we report the results. You have received written information about the study prior to this interview and have given your written consent to participate. Is that correct? [Wait for a "yes" in response from the informant] If you want to discontinue or take a break during the interview, just tell me so. Do you have any questions before we begin?”

- **Overall initial question**:

-To begin with, can you briefly tell me where you work, what professional role you have in the healthcare organisation, and in what way you get in touch with the SPAP method in regard to your professional role? …What is your (main) assignment in regard to the SPAP?

…Do you work at a central unit in the organisation or a local care unit (e.g. primary healthcare centre)? …Do you have a management position?

- **What do you know about the SPAP method?**…The SPAP method consists of several components: do you know which? …Do you know of the scientific support for SPAP?
- **What is your opinion of SPAP, as a mean for promoting physical activity among patients?**
  …Credence in the effect of the SPAP method? …What benefits does SPAP have? …What disadvantages do SPAP have? …How do you perceive the usefulness of SPAP in your professional role and in your organisation? …What is your opinion of promoting physical activity to patients in the healthcare organisation?
- **What are the procedures for prescribing SPAP within the area of your responsibility in the healthcare organisation?**

…Are there local and/or central common procedures, written routines, guidelines etcetera for: the person-centred health promotion consultation; the written prescription; follow up of the prescription; assistive devices such as pedometer or activity diary; collaboration with SPAP coordinator; collaboration with activity organisers?

- **What policy documents or guidelines do you have to follow in regard to SPAP (in your area of responsibility) in your healthcare organisation?**

…Are there written local or central guidelines/policy documents for SPAP in your healthcare organisation? …Do you have written routines for SPAP at each workplace? Are these documents easily accessible? Does these documents facilitate for using SPAP? …Is there a written work-task description for SPAP? …What does the “Hälsovalshandboken” [the Healthcare Manual] propose concerning the SPAP method?

- **How is the financial compensation for the healthcare provider expressed in the “Hälsovalshandboken” [the Healthcare Manual] concerning the SPAP method?**

…Does each workplace/healthcare unit, e.g. primary healthcare centre, get financial reimbursement (benefits) when prescribing SPAP?

- **What kind of local and/or central supporting functions/structures for SPAP are used in your healthcare organisation?**

…Are there local SPAP coordinators at each workplace/healthcare unit for coordination and development of SPAP? …Is there a centralised function within the healthcare organisation for coordination and development of SPAP?

- **Are there systems within the healthcare organisation, to provide feedback on statistics about the prescription of SPAP and how are these constructed?**

…Are statistical data on SPAP subscriptions embedded in the patient charts systems? …Who have access to extract statistics on SPAP? …Who gets statistics feedback on SPAP?

…Is it possible to get specific statistic on each SPAP component, i.e. the person-centred health promotion consultation; the written prescription; follow up of the prescription; assistive devices such as pedometer or activity diary; collaboration with SPAP coordinator; collaboration with activity organisers?

- **Does your healthcare organisation collaborate with activity organisers when prescribing SPAP?**
  …What does that collaboration look like? Activity organisers within the healthcare organisation? Activity organisers external to the healthcare organisation, e.g. Friskis&Svettis, Korpen or other sports clubs/fitness centres?

...Does the activity organiser(s) receive financial compensation from the healthcare organisation? …Does the patient get reduced rates on exercise fees financed by the activity organiser or the healthcare organisation?

- **Are there barriers for the use of the SPAP method in your healthcare organisation?**
  …At present: what factors do you see today that is impeding the use of the SPAP method at present?
  …In the future: what factors do you see today that impede (or could impede) the use of SPAP in the future?
- **Are there facilitators for the use of the SPAP method in your healthcare organisation?**
  …At present: what factors do you see today that is facilitating the use of the SPAP method at present? Has your healthcare organisation done something to facilitate the use of the SPAP method in the organization?
  …In the future: what factors do you see today that facilitate (or could facilitate) the use of SPAP in the future?
- **Do you have anything else you would like to say about the SPAP method?**

**Interview guide - prescribers of SPAP in the healthcare organisations**

**Introduction**: “The topic for this interview is the method Physical Activity on Prescription, which is usually shortened to SPAP. For more than 10 years, the method has been used in Swedish healthcare to increase health-promoting physical activity in the population. But the method has been used in many different ways in different county councils/healthcare organisations. I am interested your experiences and thoughts about SPAP from the perspective of your professional role, in which you have the right to prescribe SPAP to patients.
I will ask you some questions, the interview will be recorded, and transcribed to text after the interview. The whole interview takes about half an hour. The transcribed interviews will be coded, i.e. all information that could be linked to you will be deleted from the text. This is done to ensure that you and your answers during the interview should not be possible to recognize when we report the results. You have received written information about the study prior to this interview and have given your written consent to participate. Is that correct? [Wait for a "yes" in response from the informant] If you want to discontinue or take a break during the interview, just tell me so. Do you have any questions before we begin?”

- **Overall initial question**:

-To begin with, can you briefly tell me where you work, what professional role you have in the healthcare organisation, and in what way you get in touch with the SPAP method in regard to your professional role?

-Approximately, to how large proportion (counted in %) of your patients, respectively how many (counted in number of people) of your patients, have you prescribed SPAP to, during the previous 3 months?

- **What do you know about the SPAP method?**…The SPAP method consists of several components: do you know which? …Do you know of the scientific support for SPAP?
- **What is your opinion of promoting physical activity to patients in your professional role?**
  …Credence in the effect of physical activity on health? …As part of the professional role?

…Approximately, with how large proportion (counted in %) of your patients have been talking about physical activity in the last 3 months? …With which type of patients? …What type of physical activity do you usually talk about?

- **What is your opinion of SPAP, as a mean for promoting physical activity among patients?**
  …Credence in the effect of the SPAP method? …What benefits does SPAP have? …What disadvantages do SPAP have? …How do you perceive the usefulness of SPAP in your professional role and in your organisation?
- **What factors influences you to offer a prescription of SPAP to a patient?**
  …What factors related to the patient influences you to offer, or not to offer a prescription of SPAP?
  …What factors related to you influences you to offer, or not to offer a prescription of SPAP?
  …What factors related to the environment at your workplace influences you to offer, or not to offer a prescription of SPAP?
  …What factors related to the healthcare organisation/county council where you work influences you to offer, or not to offer a prescription of SPAP?
- **What are the procedures for prescribing SPAP at your workplace?**…Do you have written routines for SPAP at your workplace? …Are there central policies or guidelines for SPAP in your healthcare organisation?

…Are there local and/or central common procedures, written routines, guidelines etcetera for: the person-centred health promotion consultation; the written prescription; follow up of the prescription; assistive devices such as pedometer or activity diary; collaboration with SPAP coordinator; collaboration with activity organisers?
…What procedures do you follow when prescribing SPAP?

- **What support and barriers for prescribing SPAP do you see, at the local level, i.e. your workplace, and at the central level, i.e. the central healthcare organisation?**
  …How is SPAP supported by your healthcare organisation?
  …What organizational barriers and difficulties are there for SPAP?

…How is SPAP supported by your (closest) management?
…What local management barriers and difficulties are there for SPAP?
…Are there written local or central guidelines/policy documents? Are these easily accessible? Does these documents facilitate for using SPAP? Is there a written work-task description for SPAP?
…Does your workplace get financial reimbursement (benefits) when you prescribe SPAP?
…Is there a local SPAP coordinator at your workplace for coordination and development of SPAP? Is there a centralised function within the healthcare organisation for coordination and development of SPAP?
…Are there systems at your workplace and/or within the healthcare organisation, to get you feedback on statistics about the prescription of SPAP?

…Do you have suggestions on what kind of support you would like that would facilitate your prescription of SPAP?

- **Do you and/or your workplace collaborate with activity organisers when prescribing SPAP?**
  …What does that collaboration look like? Activity organisers within the healthcare organisation? Activity organisers external to the healthcare organisation, e.g. Friskis&Svettis, Korpen or other sports clubs/fitness centres?

...Does the activity organiser(s) receive financial compensation from the healthcare organisation? …Does the patient get reduced rates on exercise fees financed by the activity organiser or the healthcare organisation?

- **Are there barriers for the use of the SPAP method at your workplace or in your healthcare organisation?**
  …At present: what factors do you see today that is impeding the use of the SPAP method at present?
  …In the future: what factors do you see today that impede (or could impede) the use of SPAP in the future?
- **Are there facilitators for the use of the SPAP method at your workplace or in your healthcare organisation?**
  …At present: what factors do you see today that is facilitating the use of the SPAP method at present? Has your healthcare organisation done something to facilitate the use of the SPAP method in the organization?
  …In the future: what factors do you see today that facilitate (or could facilitate) the use of SPAP in the future?
- **Do you have anything else you would like to say about the SPAP method?**
